# Characterization of upper airway ciliary beat by coupling isolated and collective cilia motion analysis

**DOI:** 10.1186/2046-2530-4-S1-P86

**Published:** 2015-07-13

**Authors:** M Bottier, S Blanchon, M Filoche, D Isabey, A Coste, E Escudier, JF Papon, Louis

**Affiliations:** 1 Inserm UMR 955 Equipe 13, 94000, Créteil, France; 2 Université Paris-Est, Faculté de Médecine, 94000, Créteil, France; 3 CNRS ERL 7240, 94000, Créteil, France; 4 Inserm UMR 933, 75012, Paris, France; 5 Université Pierre et Marie Curie, 75012, Paris, France

## Objective

Ciliary dysfunctions may have deleterious consequences on mucociliary clearance. We propose a new approach based on coupling the isolated ciliary beat pattern and the global efficiency of ciliary beat on human ciliated cells.

## Methods

Ciliated cells issued from nasal brushing (controls and primary ciliary dyskinesia patients) were recorded by high-speed video-microscopy (350 frames s^-1^). We have performed an original quantitative analysis of ciliary beat dynamics (CBD) by following cilium tips. It allows to describe different parameters including ciliary beat frequency also measured by Fast-Fourier-Transform and Video-Kymography. We have also developed the micro-beads tracking method (MBT) to get an index of the global efficiency of ciliary beat. Here, micro-beads (4.5 µm) have been used as markers of the flow generated by beating cilia.

## Results

In term of frequency measurement, Fast-Fourier-Transform, Video-Kymography and CBD gave similar results. CBD was very helpful to discriminate controls and primary ciliary dyskinesia patients especially when ciliary beat was partially maintained. For the moment, MBT, only applied in controls, allowed to observe a flow rate from 1 up to 150 µm/s^-1 ^depending on the distance between micro-beads and beating ciliated edges (the fastest micro-beads being the closest). Interestingly, the addition of micro-beads created a stimulus that significantly increased ciliary beat frequency (~130%).

## Conclusion

Coupling Fast-Fourier-Transform, Video-Kymography, CBD and MBT is a promising approach to characterize ciliary beat under normal and pathological conditions, either congenital as primary ciliary dyskinesia or acquired as chronic rhino-sinusitis or bronchitis.

